# Biodiverse Planting for Carbon and Biodiversity on Indigenous Land

**DOI:** 10.1371/journal.pone.0091281

**Published:** 2014-03-17

**Authors:** Anna R. Renwick, Catherine J. Robinson, Tara G. Martin, Tracey May, Phil Polglase, Hugh P. Possingham, Josie Carwardine

**Affiliations:** 1 ARC Centre of Excellence for Environmental Decisions, the NERP Environmental Decisions Hub, Centre for Biodiversity & Conservation Science, University of Queensland, Brisbane, Queensland, Australia; 2 CSIRO Ecosystem Sciences, Ecoscience Precinct, Brisbane, Queensland, Australia; 3 School of Geography, Planning and Environmental Management, University of Queensland, Brisbane, Queensland, Australia; 4 CSIRO Ecosystem Sciences, Alice Springs, Northern Territory, Australia; 5 CSIRO Ecosystem Sciences, Crace, Australia Capital Territory, Australia; 6 Department of Life Sciences, Imperial College London, Ascot, United Kingdom; University of Western Australia, Australia

## Abstract

Carbon offset mechanisms have been established to mitigate climate change through changes in land management. Regulatory frameworks enable landowners and managers to generate saleable carbon credits on domestic and international markets. Identifying and managing the associated co-benefits and dis-benefits involved in the adoption of carbon offset projects is important for the projects to contribute to the broader goal of sustainable development and the provision of benefits to the local communities. So far it has been unclear how Indigenous communities can benefit from such initiatives. We provide a spatial analysis of the carbon and biodiversity potential of one offset method, planting biodiverse native vegetation, on Indigenous land across Australia. We discover significant potential for opportunities for Indigenous communities to achieve carbon sequestration and biodiversity goals through biodiverse plantings, largely in southern and eastern Australia, but the economic feasibility of these projects depend on carbon market assumptions. Our national scale cost-effectiveness analysis is critical to enable Indigenous communities to maximise the benefits available to them through participation in carbon offset schemes.

## Introduction

Climate change has focused global attention on the need to develop sustainable management responses that reduce greenhouse gases (GHG) in the atmosphere while also providing multiple benefits at local and global scales [1]. Carbon pricing mechanisms are operating in 35 countries and 13 sub-national jurisdictions, and a further seven in China are expected to start in 2013 [2]. These emission trading schemes have the potential to mitigate global climate change through altered land management practices while also providing opportunities for landholders and local communities. Tree planting, avoided deforestation and fire management are some of the many activities that are now supported by financial incentive schemes (e.g. Reducing Emissions from forest Degradation and Deforestation (REDD), and its derivative REDD+) [3]–[5]. This emerging carbon economy has the potential to cause large-scale changes in land management and trade-offs with other environmental outcomes, such as biodiversity conservation and other ecosystem services that can support community livelihoods and human well-being [6], as land uses that promote high carbon storage become more profitable [7], [8]. Climate change policy and law has developed rapidly in Australia since 2007 with the ratification of the Kyoto Protocol and the introduction of regulation and trading mechanisms for mitigating climate change. The Carbon Farming Initiative (CFI) is a carbon offsets scheme established by the Australian Government to provide farmers and other land managers with access to the voluntary and international carbon markets for reducing net carbon emissions [9]. The CFI legislation contains several provisions to align carbon projects with local sustainable development objectives, including provisions to promote projects that provide benefits for biodiversity or Indigenous communities. Globally, many Indigenous leaders have advocated that Indigenous community participation in carbon offset projects may offer negotiated and long-term support to meet and sustain local Indigenous community livelihoods, and cultural, social and economic development aspirations [10]–[13]. Although the nature of Indigenous carbon rights varies in time and space across Australian jurisdictions there is considerable interest from Indigenous communities in the extent and location of the range of co-benefits (direct sustainable development outcomes associated with a carbon offset project that are additional to emissions avoided or carbon stored).

Robinson et al. [12] consulted with Indigenous leaders and organisations across Australia and found that Indigenous people are interested in participating in carbon markets and carbon offset strategies with their associated co-benefits to receive direct carbon payments and also pursue a range of other opportunities including working on country, education and training, and enhanced decision-making power for their traditional estates. Prospective Indigenous carbon co-benefits may also be pursued on land without Indigenous land tenure if Indigenous people are employed to manage carbon offset projects and associated ecosystem services. The co-benefits that carbon offset projects might offer local Indigenous people and broader society could include the delivery of ecosystem services and biodiversity benefits in addition to generating carbon credits. This paper provides a spatial analysis of the carbon and biodiversity potential of one offset method, planting biodiverse native vegetation, on Indigenous land across Australia. Mixed trees plantings store at least, or even greater, amounts of carbon as monocultures and also have the benefit of providing greater resistance and resilience to disturbances, and the provision of other ecosystem services [14].

## Methods

**Figure 1:**
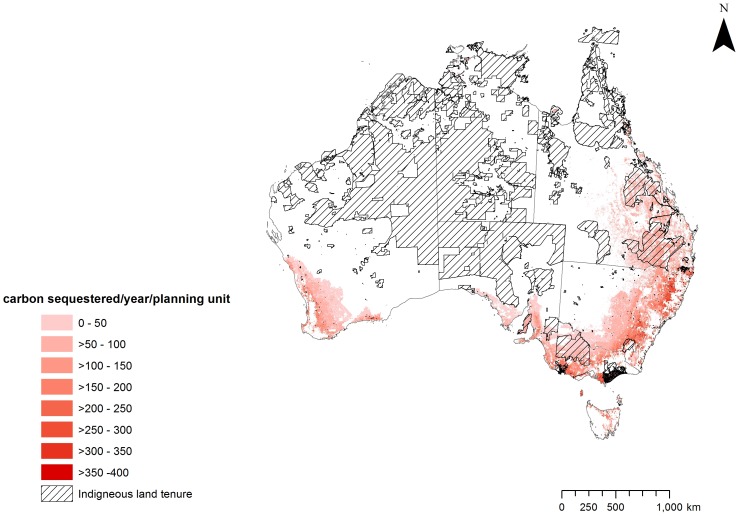
Map of Indigenous tenure overlaid on plantable areas for biodiversity.

In Australia, extensive land clearing has occurred since the industrial revolution. We therefore restricted our study to areas that were historically (pre-1750) covered by a vegetation type containing trees of at least 1.3 m tall but have since been cleared of this native vegetation, excluding built areas. We refer to this as the ‘potential area for biodiverse planting’ (Figure 1). As this study focuses on tree planting for carbon and biodiversity benefits, we assumed that the species planted would be consistent with pre-existing vegetation types [15].

We compiled a comprehensive map of Indigenous tenure across Australia. Indigenous land tenure data was sourced from the official agencies responsible for the registration of various Indigenous land tenures including the National Native Title Tribunal, Conservation and Protected Areas Database, Australian Government Department of Sustainability, Environment, Water Population and Communities, Indigenous Land Corporation, National Land and Water Resources Audit and Geoscience Australia. The classes of Indigenous land represent legally recognised tenure, and they vary in the type of rights and opportunities conveyed with the form of tenure. Table 1 documents the data sources, classes and description of tenure used to create the map.

**Table 1:** Methodology for displaying the Indigenous Estate in Australia: The cadastral position of boundaries should be regarded as approximate.

Layer	Date	Tenure	Layer Description and lineage	Data Source
Indigenous Protected Areas	26/07/12	Type of reserve = Indigenous Protected Area; Status of agreement = Declared.	Indigenous Protected Areas that have been declared by the Australian Government, through the implementation of the Indigenous Protected Areas Programme.	Australian Government Department of Sustainability, Environment, Water, Population and Communities. http://www.environment.gov.au/indigenous/ipa/index.html
Indigenous Land Use Agreement	2/08/12	Agreement Type = Area Agreement , Body Corporate; Agreement Status = Registered	Indigenous land use agreement that have been registered with the National Native Title Tribunal.	Created by the National Native Title Tribunal in 1998 and continuously updated and maintained. Should at all times reflect the primary detail as contained within the Register of ILUA's. Download from Geoscience Australia. ANZCW0703011415
Native Title Determinations Register	17/06/10	Determination Outcome = Native title exists in parts of the determination area OR Native Title exists in the entire determination area	Boundaries and information about each determination of native title. Native title exists in parts of the determination area OR Native Title exists in the entire Determination area.	Created by the National Native Title Tribunal in 1994 and continuously updated and maintained. Download from Geoscience Australia. ANZCW0703011416
Collaborative Australian Protected Areas Database (CAPAD) 2010 - External	1/07/10	Type of CAPAD reserve = Aboriginal Area, National Park Aboriginal, Nature Park (Aboriginal)	Selected Indigenous values from “The Collaborative Australian Protected Areas Database” (CAPAD) which provides a snapshot of protected areas that meet the IUCN definition of a protected areas for Continental Australia.	Compiled by the Department of Sustainability, Environment, Water, Population and Communities, from information supplied by State and Territory conservation agencies. Downloaded from the DIG website http://www.environment.gov.au/parks/nrs/science/capad/index.html.
Indigenous Land Corporation	19/10/11	Holding Status = Tenure granted and Tenure held	Layer is generated by intersecting x,y coordinates of the centroid of tenure parcels supplied by the Indigenous Land Corporation and the PSMA National Land Tenure Classification.	Compiled layer by T.May. Base layers: Indigenous Land Corporation Tenure held and granted and PSMA Australia National Land Tenure Classification Version 1.2
Land Tenure in Australia Rangelands (1955 to 2000)	1955–2000	tenure 1999 = Indigenous Tenure (IND_T), Aboriginal land trusts, land councils or Aboriginal Local Governments OR tenure 1999 = Indigenous Lease (IND_L) - Pastoral leases issued to indigenous entities.	This data set describes the land tenure across Australia's rangelands between 1955 and 2000. IND_T represents Indigenous land that was administered by the Crown (States) until the 1970s for WA, SA, and NT, 1980s for NSW, and 1990s for QLD. Thereafter Aboriginal land trusts, land councils or Aboriginal Local Governments assumed responsibility. IND_L represents pastoral leases issued to indigenous entities. A subset has been created using the IND_T and IND_L attributes in the Tenure type 1999 field of the data set.	National Land and Water Resources Audit (NLWRA)
Australian Land Tenure 1993	1993	Feature Land tenure categories over 100 km^2^, Aboriginal reserve, Aboriginal freehold, Aboriginal leasehold and Aboriginal freehold-national park.	Australian Land Tenure 1993 has been derived from Geoscience Australia's National Public and Aboriginal Lands data and supplemented with additional information. A subset of Aboriginal lands comprising private leasehold, freehold and reserves held by or on behalf of Aboriginal communities has been extracted from the original polygon data source.	Geoscience Australia. © Commonwealth of Australia, 2004. www.ga.gov.au. ANZCW0703005424
National Land Tenure Classification	2008	Base layer for creation of the Indigenous Land Corporation intersect.	PSMA Australia National Land Tenure Classification Version 1.2. PSMA Australia National Land Tenure Classification Version 1.2. The National Land Tenure Classification is a dataset showing the Tenure Type, both freehold and non-freehold, for land parcels in Australia.	PSMA Australia National Land Tenure Classification Version 1.2. PSMA Australia Limited. www.psma.com.au
Australian Boundary	2004	All attributes displayed	Coastline and state borders for Australia.	Geoscience Australia. © Commonwealth of Australia, 2004. www.ga.gov.au

**Figure 2:**
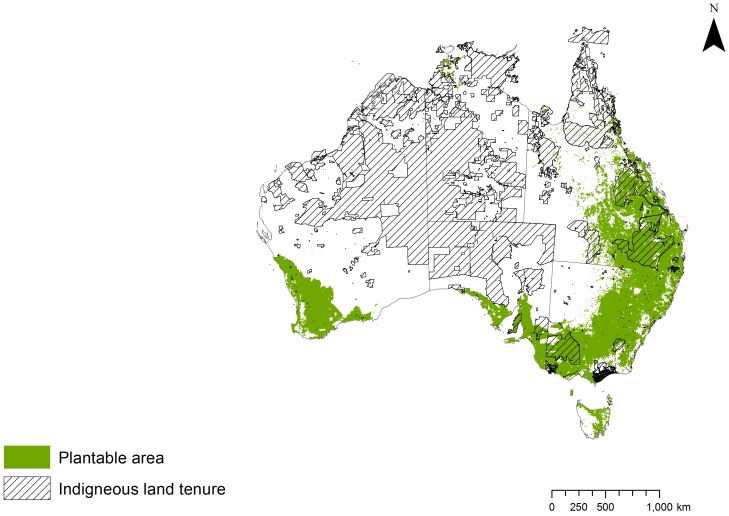
Map of Indigenous tenure overlaid on carbon sequestered (tonnes) per year per planning unit (4 km^2^) in plantable areas.

The potential carbon that could be sequestered in our study area by growing trees was estimated using data on carbon sequestration potential for mixed environmental tree plantings from Polglase et al. [16] (Figure 2). Carbon sequestration rates per year, averaged over a 40 year period at a resolution of 1 km2, were estimated from the 3-PG2 model of tree growth using data on monthly climate data, site factors, initial stocking rate and management conditions. A 40 year period was chosen as above that sequestration rates are considered negligible. The model was calibrated and validated against sites for environmental plantings, primarily in south-eastern Australia and lower rainfall zones (<800 mm). Detailed methodology on calculating rates of carbon sequestration can be found in Polglase et al. [16].

We used vegetation types as surrogates for determining the potential of carbon planting to produce biodiversity co-benefits. The 63 major vegetation subgroups from the National Vegetation Information System (NVIS version 3.1) were intersected with the 85 Interim Biogeographic Regionalisation of Australia (IBRA) bioregions to generate 1886 unique vegetation types across Australia. Of these, 1185 vegetation types contained trees of at least 1.3 m tall and thus qualified for carbon planting. Using the pre-1750 and current extent of these vegetation types, a total of 139 vegetation types (within 32 major vegetation subgroups) have been cleared to below 30% of their pre-1750 extent and are therefore a high priority for restoration. Targets were set at the number of hectares required to increase each vegetation type up to 30% of original (pre-1750) extent. Restoration of these vegetation types to the targets set was considered as a biodiversity co-benefit of carbon planting. The pre-existing extent of one vegetation type (melaleuca open forests on Victorian volcanic plains) is now largely covered by built-up areas therefore it is not possible to restore it to 30% and thus its maximum possible restorable area was set at 11% of original extent.

The cost-effectiveness of locations for meeting vegetation restoration targets (restoration of each vegetation type up to 30% pre-1750 extent or maximum extent possible if the 30% target could not be met) and carbon sequestration across Australia at a resolution of 4 km^2^ were determined by Carwardine et al. (unpublished data) using the conservation planning tool Marxan (version 2.43) [17]. An economic scenario was chosen to reflect the Australian government's 2011–2013 carbon price of AU$23/tonne (establishment cost AU$1000 per ha, no water cost, baseline growth rate, discount rate 5%, carbon price AU$20/tonne which is slightly lower that the trading price to account for transaction costs).

A grid-based layer containing 99,190 planning units of 4 km^2^ over the potential area for biodiverse planting across Australia was created. The extent of each vegetation type in each planning unit was determined using Spatial Analyst in ArcMap version 10. Each planning unit was assigned the average sequestration rate and profitability under the economic scenario described above and multiplied by the plantable area in that planning unit to give the total potential carbon sequestered per year and the potential profitability for biodiverse carbon planting. Marxan uses a simulating annealing algorithm to identify alternative sets of planning units to meet the biodiversity targets and sequester carbon at a minimum cost. Marxan was set to generate 500 alternative area sets to meet the targets and the selection frequency of each planning unit was used as a measure of its relative priority for meeting this combined goal cost-effectively, with those selected a higher number of times having a higher priority.

Using ArcMap version 10 we overlaid the map of Indigenous land tenure with maps of carbon sequestration, biodiversity features, and relative priority areas, in order to determine (i) the proportion of carbon and biodiversity goals that can be met on Indigenous land and (ii) the proportion of priority areas (in categories of very high, high, moderate, low) that overlies Indigenous land.

## Results

Approximately 92 million hectares (Mha) of land in Australia has been cleared and has the potential to be replanted or regenerated with native forests and woodlands (total biodiversity plantable area). This would sequester 710Mtonne CO_2_ per year in the short term. However, only part of this area would be profitable for environmental planting depending on the carbon price and other economic assumptions, and only a proportion of it would be available due to many other social and practical constraints [16]. Using the scenario reflecting Australia's 2011–2012 carbon trading price of AU$23/tonne , 31Mha of this area is potentially profitable for carbon farming with the capacity to sequester 294 Mt CO_2_ [16]. Indigenous land covers 9.7Mha of this area, and has the theoretical potential to sequester 83 Mt CO_2_ (28% of the total) (Figure 2). A carbon price of AU$5/tonne which is close to the 2013 price on the international markets would reduce the profitable area for carbon planting to 5Mha and the potential for Indigenous communities to be involved would be severely limited.

To assess the potential biodiversity co-benefits that could be generated through biodiverse plantings on Indigenous land we used targets of restoring each heavily cleared native vegetation type to at least 30% of its pre-1750 extent. Forty-eight per cent of the total biodiversity plantable area contains 139 vegetation types that have been cleared to less than 30% of their pre-1750 extent. Of the total biodiversity plantable area, 17Mha (19%), containing 79 vegetation types, is on Indigenous land, primarily in southern and eastern Australia (Figure 1). In total, the targets for 18 vegetation types can be met solely on Indigenous land and two of these vegetation types (casuarina and allocasuarina forests and woodlands, and melaleuca open forests and woodlands) can only be restored in a natural ecosystem on Indigenous land.

**Figure 3:**
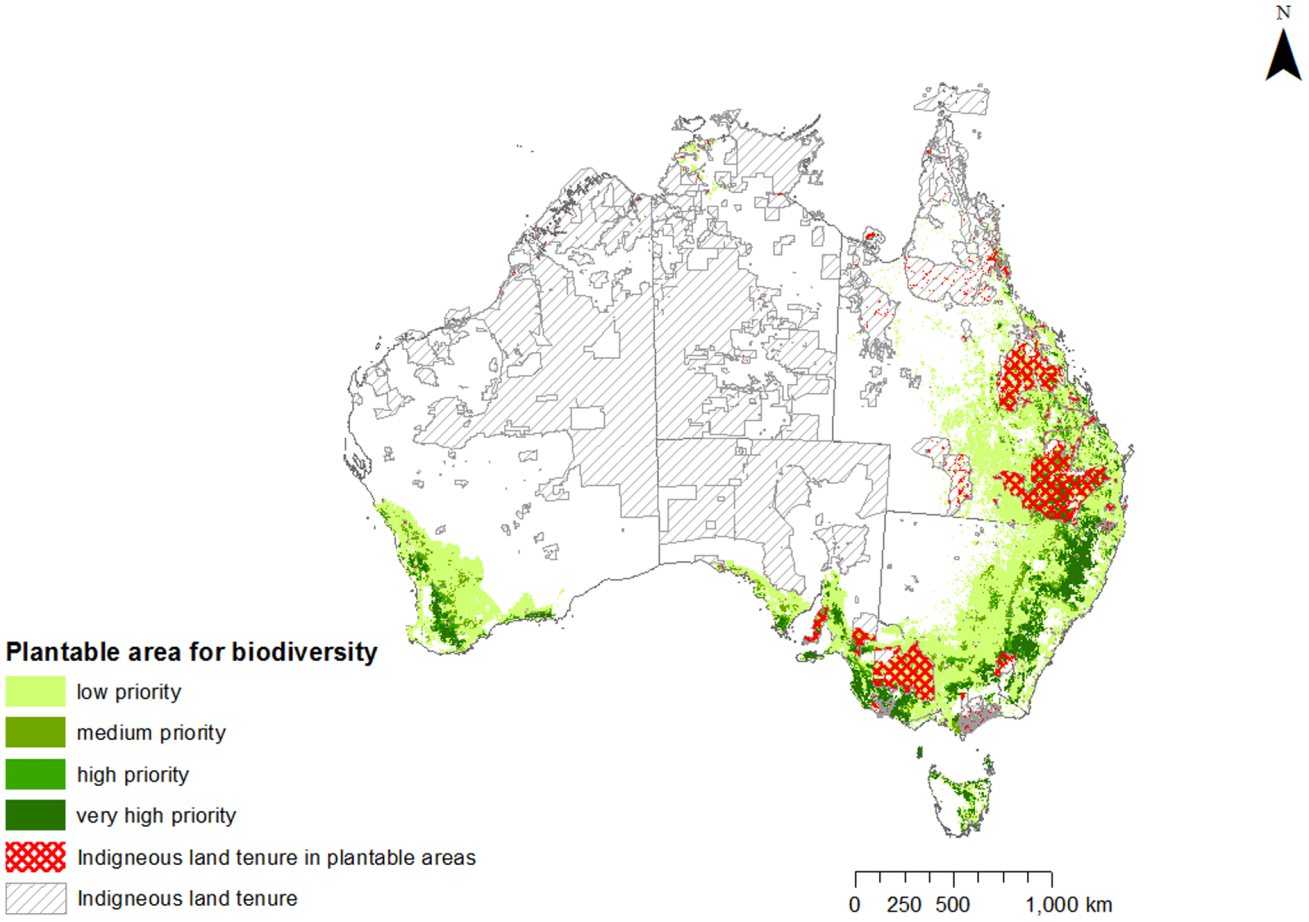
Relative priority (selection frequency) of areas for meeting biodiversity and carbon goals for environmental planting overlaid with Indigenous tenure.

Over 16% of the very high priority areas (75–100% selection frequency) for cost-effectively meeting both carbon and biodiversity goals are on Indigenous land, primarily in southeast Queensland, eastern New South Wales, southwest Victoria, southeast South Australia and southwest Western Australia. This percentage is higher in high (50-<75% selection frequency) and medium (25-<50% selection frequency) priority areas (Figure 3; Table 2). Almost 19% of the combined high and very high priority areas for cost-effectively meeting biodiversity goals are on Indigenous lands. Given that Indigenous land cover 19% of the total biodiversity plantable area, this result indicates that Indigenous lands are approximately as important as the rest of Australia for achieving this combined goal cost-effectively.

**Table 2:** Carbon and biodiversity goals met in all plantable areas on land under Indigenous tenure.

	Total plantable area	Area of Indigenous Tenure (with the % of the attribute in the total plantable area)
Total plantable areas	92Mha	17Mha (18.9% of total plantable area)
Carbon CO_2_^−e^ seq. in plantable areas	710 Mt/year	145 Mt/year (20.5% of total carbon sequestered in plantable areas)
**Relative priority for tree planting to meet biodiversity goals**		
Very high priority areas (75–100%)	17.2Mha	2.8Mha (16.1% of very high priority areas)
High priority areas (50-<75%)	7.6Mha	1.9Mha (24.4% of high priority areas)
Medium priority areas (25-<50%)	11.2Mha	2.6Mha (23.5% of medium priority areas)
Low priority areas (0-<25%)	56.5Mha	10.2Mha (18.1% of low priority areas)

## Discussion

These results show that carbon offset schemes such as those available through Australia's CFI offer a potentially important opportunity to deliver carbon and biodiversity benefits on Indigenous lands. Our analyses show that the most cost-effective areas for achieving these benefits from biodiverse planting are located primarily in southern and eastern Australia. There are less carbon and biodiversity benefits from planting on Indigenous lands in Australia's northern regions. This finding reflects the low density of forests and low level of historical clearing in northern Australia. Importantly, Indigenous communities in Australia and around the world are faced with a wide range of carbon offset schemes that may benefit their local sustainable development objectives. For example, some Indigenous communities in Northern Australia are reducing greenhouse gas emissions through an approved early dry season savanna burning carbon offset scheme. There is also a rapid growth in voluntary markets now available to Indigenous landholders and local communities and each have standards regarding the contribution of these offset schemes to sustainable development (www.climate-standards.org/).

The rapid growth of carbon markets internationally has been accompanied by extensive criticism of the contribution of these markets to sustainable development, particularly in terms of providing co-benefits to local Indigenous communities [18]. For example, there is concern that carbon forestry will result in the loss of traditional Indigenous livelihoods and land-use practices [19], and that the spiritual and natural values of the forests will not be valued [20]. Growth in voluntary market volumes has been variable and far from certain and the fluctuating price of carbon credits can pose real challenges to the long-term viability of carbon offset schemes [21]. A framework to meet the minimum standards to protect and include Indigenous people's rights in government carbon policies has been established by the United Nations Declaration on the Rights of Indigenous Peoples. The aim of this is to ensure that Indigenous people have appropriate involvement in the development of carbon markets that impact their land [13].

There are caveats to our approach which need to be highlighted. The carbon sequestration model was calibrated using data primarily from southern temperate zones and may not be as applicable to northern, wet and arid parts of Australia. However, as we restricted our analyses to only the potentially plantable areas for forest and woodland types, much of northern, central or arid areas were not included. The value of land over biodiversity plantable areas was used as a surrogate for the opportunity cost of changing land use to carbon farming. However the analysis assumes that carbon planting projects are undertaken without buying and selling land. It is also important to note that many areas assessed as ‘very high priority’ for delivering biodiversity benefits through carbon plantings are marginally profitable based on carbon payments alone. In order to determine the feasibility of Indigenous carbon projects, which could be subject to unique establishment costs (e.g. training and community engagement needs) and information needs (e.g. where and what revegetation schemes are possible and desirable), model results will need to be tested in partnership with local Indigenous communities [22]. In addition, the recent change in government in Australia highlights the instability of the policy and political environment surrounding the CFI opening up new risks and uncertainties to carbon farming efforts, including those adopted by Indigenous communities.

A spatial cost-effectiveness analysis of the carbon and biodiversity potential of biodiverse planting on Indigenous lands offers valuable higher level information to assist Indigenous and non-Indigenous collaborators globally to consider potential carbon planting projects. Only through such an analysis can we evaluate the benefits or opportunities that may be achieved through environmental plantings on a national scale. This is an essential input for identifying opportunities for culturally appropriate ways to generate carbon credits that contribute to biodiversity and other benefits to Indigenous people and broader society.
